# Positive Predictive Factors for Urogenital Injuries in Severely Injured Patients with Pelvic and Spinal Fractures: Introducing the UPPS Scoring System

**DOI:** 10.3390/medicina58111583

**Published:** 2022-11-02

**Authors:** Olivia Anna Mair, Maren Himmler, Suna Brunnemer, Christoph Faymonville, Patrick Honeck, Thomas Horn, Peter Biberthaler, Marc Hanschen

**Affiliations:** 1 Department of Trauma Surgery, Technical University of Munich, Klinikum rechts der Isar, Ismaninger Strasse 22, 81675 Munich, Germany; 2 Department of Urology and Urological Surgery, University Medical Center Mannheim, Theodor-Kutzer-Ufer 1-3, 68167 Mannheim, Germany; 3 Department of Trauma Surgery, University Medical Center Mannheim, Theodor-Kutzer-Ufer 1-3, 68167 Mannheim, Germany; 4 Department of Urology, Technical University of Munich, Klinikum rechts der Isar, Ismaninger Strasse 22, 81675 Munich, Germany

**Keywords:** urogenital injuries, polytrauma, scoring system, predictive factors

## Abstract

*Background and Objectives*: Although urogenital injuries are common in severely injured patients, their diagnosis is often delayed. Predicting genitourinary injuries (GUI), especially in the immediate stages post injury, remains a challenge. This study aims to evaluate and determine positive predictive factors for the presence of GUI in polytrauma patients. Subsequently, these factors shall be used to develop an easy-to-use scoring system, deployable directly in the emergency setting. *Materials and Methods*: This study evaluates all severely injured patients with an Injury Severity Score (ISS) ≥ 16 admitted to the emergency departments of two German university hospitals between 2016 and 2020. These patients were retrospectively scanned for injuries of the thoracic and/or lumbar spine and/or the pelvic girdle. Demographic data was analyzed alongside trauma mechanism, type of injuries, mortality, length of hospital stays, surgeries, laboratory results, and urological treatment. Subgroup analysis was performed to compare patients with and without GUIs using *t*-tests. Conducting a binary logistic regression model, the significant factors were combined to create a scoring system, which was further analyzed for accuracy. *Results:* In total, 413 patients with an average ISS of 33.8 ± 15.0 were identified, and 47 patients (11.4%) sustained urogenital injuries with an average Abbreviated Injury Scale (AIS) score of 2.3 ± 1.1 (range: 1–5). The severity of the pelvic girdle injury correlated with the presence of urogenital injuries (*p* = 0.002), while there was no correlation with spinal injuries. Moreover, most GUIs resulted from motorcycle accidents (*p* < 0.001) and 87.2% of these patients were male. Patients with GUI were significantly more likely to show macrohematuria (*p* < 0.001) on admission and were more severely injured overall (ISS > 34). There was no significant difference in the length of intensive care unit (ICU) stay, the days until discharge, or death rates. *Conclusions:* Factors or circumstances which reliably predict the presence of GUI were found to include the male sex, a motorcycle accident, high severity of pelvic girdle fractures, macrohematuria on admission to the emergency department, and an ISS > 34. With these findings, we introduce the ‘Urotrauma in Polytrauma patients with Pelvic and/or Spinal injuries’ (UPPS) score for easier prediction of GUI in the emergency setting.

## 1. Introduction

Trauma-related injuries remain the leading cause of death in industrialized countries for adults under the age of 45 [1]. Sustained injuries and the associated, poor long-term outcomes after polytrauma impact the patients’ capacity to work and thus cause a large socioeconomic burden [2]. Genitourinary injuries (GUIs) occur in approximately 5–10% of polytrauma patients, with traumatic kidney injuries accounting for the largest proportion [3,4]. It can be difficult to identify GUIs in severely injured patients during the initial emergency response. Therefore, delayed or even completely missed diagnoses of GUIs present a major challenge in the management of polytrauma patients. There have been attempts to predict GUIs with identifying markers such as macrohematuria. While often associated with GUI, macrohematuria has been shown to be unreliable as a sole marker, due to the number of patients without it who still have urogenital injuries and its inability to show the severity or the location of the injury in the genitourinary tract [5,6,7]. 

In industrialized countries, GUIs are mainly caused by blunt abdominal trauma, whereas in developing countries, penetrating trauma such as stabbing or gunshot wounds is the leading mechanism of injury [8]. Furthermore, lower urinary tract injuries (LUTI), particularly urethral injuries, are very commonly (up to 83%) associated with pelvic girdle fractures or acute deceleration trauma such as high-speed traffic accidents or falls from great heights [9,10]. 

Although traumatic GUIs are not usually the cause of death initially, they can significantly affect the quality of life and lead to high rates of morbidity and mortality in the recovering process after polytrauma [9,11,12]. Therefore, it is critical to identify the presence of GUIs as soon as possible to prevent complications or simply delay in treatment.

The obligatory computed tomography (CT) scan at the initial presentation of a polytrauma patient to the trauma room usually does not include explicit imaging of the urogenital organs, such as a urographic phase [8,13]. Overall, the symptoms of GUI are quite vague and therefore can be easily overlooked, especially in patients with more obvious and pressing injuries. On the other hand, pelvic fractures and spinal injuries are far more likely to be spotted on the initial CT scan. Due to the close anatomical relationship between the pelvic girdle and the lower urinary tract, injuries in this body region can point towards the presence of GUIs [14]. Injuries to the thoracic and/or lumbar spine are theorized to link to a higher risk of GUI, but this has not yet been exhaustively evaluated. 

As the problem of diagnosing GUIs in severely injured patients is well known, several authors have attempted to identify predictive factors for GUIs in polytraumatized patients [5,7,10,14,15]. To our knowledge, no clear criteria for the prediction of GUIs in severely injured patients with pelvic and/or lumbar and/or thoracic spinal injuries have been identified to date. 

Therefore, this study aims to determine predictive factors to anticipate GUI in severely injured patients. Another aim is to facilitate decision making in the trauma room to avoid delayed diagnosis of GUI and subsequent incorrect treatment in polytrauma patients. To make this decision process easier, we will later present a novel and easy-to-use scoring system. 

## 2. Materials and Methods

We conducted a multicenter, retrospective observational cohort study of trauma admissions to two Level-1 trauma centers in Germany. The study was approved and authorized by the Institutional Review Boards (Hospital 1: 229/20 S-EB, approved on 20 April 2020); Hospital 2: 2020-835R, approved on 25 May 2020) and is registered under NCT05074095. 

All patients admitted to the trauma room during the 5-year study period (2016–2020) were retrospectively analyzed. We included all patients with an Injury Severity Score (ISS) ≥16 and injuries of the pelvic girdle and/or the thoracic and/or lumbar spine. Excluded were patients under 18 years of age, pregnant women, patients with missing data, and patients with a total ISS <16. Data collection was performed simultaneously at the two participating hospitals. 

Data was collected in analogy to the TraumaRegister DGU^®^ data entry form in the version V2020 [16]. We collected patient demographic data, the American Society of Anesthesiologists score (ASA) before accident and all diagnoses. In terms of injury, we distinguished between penetrating and blunt trauma. More specific information was provided on traffic accidents (car/truck occupant, motorcyclist, bicycle rider or pedestrian), falls (from a height of more or less than 3 m) and other forms of injury (stabbing, gunshot wounds or other blunt trauma). The Abbreviated Injury Scale (AIS) was determined for all injuries using the Association for the Advancement of Automotive Medicine (AAAM’s) AIS© 2005 Update 2008 Manual. The AIS is an anatomically based scoring system, which classifies injury severity in each body region on a scale from 1 to 6: 1: minor injury, 2: moderate, 3: serious, 4: severe, 5: critical, and 6: maximal. The AIS scores were then used to calculate the Injury Severity Score (ISS) as per definition. The AO-Classification was noted for all pelvic and spinal fractures. In case of multiple fractures in one category, the fracture with the highest AIS score was noted (according to the Maximum Abbreviate Injury Scale (MAIS) principle). 

Additionally, we collected information on all surgeries, time of surgery, length of hospital- and intensive care unit (ICU)-stay, time of death (if applicable) and specific urological data. More specifically, we analyzed glomerular filtration rate (GFR) and creatinine levels at the time of admission to the emergency department (ED), day 3/4 and day 7 after trauma, hematuria, oliguria and anuria documented in the digital patient record and all urological diagnostics, procedures and surgeries with the exact timing. Both participating hospitals follow the Advanced Trauma Life Support^®^ (ATLS^®^) guidelines for treatment of severely injured patients.

All data was collected anonymized using Microsoft^®^ Excel^®^ for Mac 2011 (Microsoft Corporation, Redmond, WA, USA). Statistical analysis was performed using IBM^®^ SPSS^®^ Statistics Version 26 (International Business Machines Corp., New York, NY, USA). First, we compared the data of the two participating hospitals to verify data comparability. Thereafter, we analyzed demographic patient data and evaluated predictive factors for the prevalence of GUIs. Statistical significance levels were two-sided and *p*-values <0.05 were considered statistically significant. Patients’ characteristics were described using mean ± standard deviation (SD) for continuous variables and median with interquartile range (IQR) for variables without normal distribution. Absolute and relative percentage frequencies were used for categorical variables. Significances were calculated using unpaired two-sample *t*-tests for continuous variables and Chi-Square Test for categorial variables.

Secondly, we used the factors which showed high (*p* < 0.05) to very high (*p* < 0.001) predictive value to create a novel scoring system. We combined these factors together utilizing a binary logistic regression model. Factors with high (*p* < 0.05) predictive value were attributed single points towards the scoring system, factors with very high (*p* < 0.001) predictive value were attributed double points towards the scoring system. We further generated a receiver operating characteristic (ROC) curve to evaluate the utility of the proposed scoring system and an area under the curve was calculated.

## 3. Results

In total, 413 patients with a mean age of 52.4 ± 20.4 years were included in this study (Hospital 1: 215 patients; Hospital 2: 198 patients); 28.8% (*n* = 119) of our study group were female and 71.2% male (*n* = 294). The mean ASA score before the accident was 1.7 ± 0.7, and the median ISS was 29 (IQR 22–41). Urogenital injuries were detected in 47 (11.4%) patients, and the mean AIS score of these urogenital injuries was 2.3 ± 1.1. We recorded 19 (40.4%) renal injuries (AIS 3.1 ± 1.0), 3 (6.4%) bladder injuries (AIS 1.7 ± 0.6), 11 (23.4%) urethral injuries (AIS 2.7 ± 0.7) and 14 (29.8%) injuries of the external genitalia (AIS 1.4 ± 0.6). The most common mechanism of injury was high fall (≥3 m) (*n* = 106, 25.7%), followed by car/truck accidents (*n* = 80, 19.4%) and motorcycle accidents (*n* = 70, 16.9%). One patient’s mechanism of injury could not be determined as the patient was found on the street unconscious and died later. Table 1 shows a comparison of the data between the two level-1 trauma centers. Demographics were very similar when comparing the participating hospitals, except for slight age-differences, which we believe does not have an impact on the overall results.

### 3.1. Trauma Mechanism

In order to investigate whether the mechanism of trauma influences the prevalence of genitourinary injuries, we analyzed each mechanism of trauma individually. The results showed a statistically significant higher rate of GUIs in motorcycle accidents (*p* < 0.001) compared to all other mechanisms of injury. No other mechanism of injury resulted in statistically significantly more urogenital injuries. Although both high and low falls were statistically significant (*p* = 0.033, *p* = 0.009, respectively), they cannot be used as a predictor for GUIs as they more often led to injury patterns without GUIs.

### 3.2. Further Predictive Factors for Urogenital Injuries

In addition to the above findings, male patients were more likely to sustain urogenital injuries than females (*p* = 0.010), as 41 (87.2%) of the 47 urogenital injuries were found in males. When comparing the likelihood of GUIs in patients with a total ISS >34 to patients with an ISS ≤34, more severely injured patients were significantly more likely to sustain GUIs (median 35 (IQR 21–50) vs. median 29 (IQR 22–38.75), *p* = 0.032).

Patients with and without urogenital injuries had comparable death rates (10.6% vs. 15.3%, *p* = 0.514). Moreover, urogenital injuries did not prolong hospitalization (32.2 ± 27.9 vs. 27.0 ± 24.5 days, *p* = 0.184) or lead to longer ICU treatment (13.4 ± 18.2 vs. 12.5 ± 16.8, *p* = 0.668). 

Table 2 summarizes the patient characteristics and significant findings between the groups with and without urogenital injuries.

### 3.3. Spinal Trauma and Urogenital Injuries

In total, 109 (26.4%) patients sustained injuries to the lumbar and 109 (26.4%) patients to the thoracic spine; 53 (12.8%) patients sustained injuries to the lumbar as well as the thoracic spine. The mean AIS was 2.6 ± 0.9 for thoracic spine injuries and 2.4 ± 0.7 for lumbar spine injuries. No significant correlations were found between thoracic or lumbar spine injuries and the prevalence of urogenital injuries. Furthermore, the severity of injuries also had no effect on the prevalence of urogenital injuries.

### 3.4. Fractures of the Pelvic Girdle and Urogenital Injuries

We recorded 237 (57.4%) pelvic fractures with a mean AIS of 3.4 ± 1.1. A total of 39 (16.5%) patients with pelvic injuries also sustained GUIs and 83% (39/47) of all GUIs occurred in patients with pelvic injuries. Therefore, significantly (*p* = 0.002) more patients sustained urogenital injuries when pelvic fractures were present. Additionally, more severe injuries of the pelvic girdle made the likelihood of urogenital injuries much higher (*p* < 0.001). No significant differences were found when comparing the likelihood of pelvic or spinal fractures between the different trauma mechanisms. 

### 3.5. Urological Findings

No significant differences in GFR and creatinine levels were observed in patients with or without urogenital injuries at admission, day 3/4, and day 7 after admission (*p* = 0.262, *p* = 0.119, *p* = 0.267, respectively). Significantly more patients showed macrohematuria at admission when urogenital injuries were present (38.3% vs. 1.9%, *p* < 0.001). In total, 48.0% of patients who received CT urography actually had urogenital injuries. 

### 3.6. The ‘Urogenital Trauma in Polytrauma Patients with Pelvic and/or Spinal Injuries’ (UPPS) Scoring System 

By adding all of the aforementioned highly predictive factors together we were able to create the novel “Urogenital trauma in Polytrauma patients with Pelvic and/or Spinal injuries” (UPPS) scoring system. 

**Figure 1:**
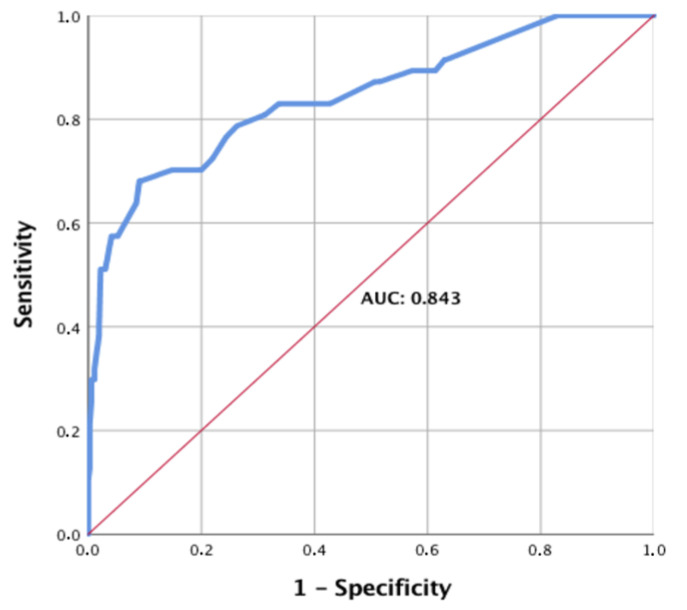
ROC (receiver operating characteristic) for predicting urogenital injuries in polytrauma patients. The ROC (receiver operating characteristic) for use of the above scoring system in predicting urogenital injuries in polytrauma patients, the AUC (area under the curve) is 0.843.

To evaluate the strength of this model, a receiver operating characteristic (ROC) curve was generated (Figure 1). 

**Figure 2:**
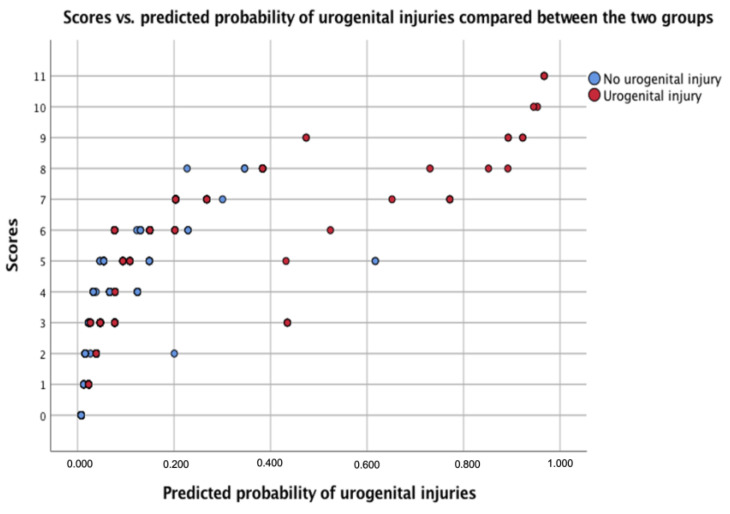
Assessment of the proposed UPPS score. Scatter blot demonstrating UPPS score vs. predicted probability of urogenital injuries, compared between patient groups with and without urogenital injuries.

The area under the curve (AUC) was 0.843 with a confidence interval of (0.77; 0.91), showing high prediction strength. Additionally, an assessment of the scoring system was performed, comparing the prediction with the actual scores (Figure 2). 

Herein, we introduce the novel UPPS score to facilitate decision making in the emergency setting and to provide the treating physicians with an easy-to-use tool (Table 3). The maximum score that can be achieved is 11 points. Scores between 7 and 11 make the presence of a GUI very likely. With a score between 4 and 6, the risk of urogenital injuries is moderate and should always be considered. A score of 0–3 makes the presence of urogenital injuries unlikely. Further, we evaluated the test’s value by calculating the sensitivity and specificity. Specificity was defined as the percentage of patients with low scores (0–3 points), who truly did not have a GUI. Sensitivity was defined as the percentage of patients with higher scores (>3 points), who truly had GUI. The test therefore has high negative predictive value with high specificity (93.4%), yet only medium to moderate sensitivity (55.4%).

## 4. Discussion

Managing polytrauma patients requires timely diagnosis of GUIs, yet this is not always given, nor is it a major focus for research. To our knowledge, this study is one of the most comprehensive in this field. 

In total, 11.4% of our patients sustained urogenital injuries, which is slightly more than the prevalence of 5–10% described in the literature [3,4,8,15]. On one hand, this could be due to the fact that we included all GUIs and did not distinguish between severe and light GUIs (as measured by the AIS scores) [8]. On the other hand, this high prevalence of urogenital injuries could also be explained by our inclusion criteria, as we included only patients with pelvic fractures and/or injuries to the thoracic and/or lumbar spine. Nevertheless, we acknowledge that these inclusion criteria could also lead to selection bias in terms of a higher overall percentage of GUIs in our study population. Our study shows that patients with pelvic girdle injuries are more likely to sustain urogenital injuries, which has been demonstrated in the literature as well [11,14,17]. We did not find any correlation between GUIs and injuries of the thoracic and/or lumbar spine, which, to our knowledge, has not been evaluated before. A possible explanation could be that the thoracic and lumbar spine, although anatomically close, are not directly ligamentously connected to the urogenital organs, especially the kidney and the ureter, as is the case with the urethra and the pelvic ligaments and girdle. Most other studies have so far only analyzed patients after polytrauma in general. We believe narrowing the patient cohort down to patients with injuries in close anatomical proximity to the genitourinary tract, will lead to more precise diagnosis of GUI.

In addition to the correlation of pelvic fractures with GUI in general, our data confirms the importance of the severity of the pelvic ring injury. This is shown by the likelihood of sustaining a GUI, which correlates directly with the severity of the pelvic girdle fracture as measured by the AIS score. AIS scores for pelvic fractures are high (AIS 4/5) when the pelvic ring is completely unstable, as is the case with vertical shear and open book fractures, or when there is a concomitant major blood loss [14,18]. This confirms the findings of prior studies in which pelvic fracture related urogenital injury (PFUI) was mostly associated with widening of the symphysis and shear forces in fractures/ dislocation of the sacroiliac joint with disruption of the anterior pelvic ring. Nevertheless, as a sole predictor the presence of a pelvic girdle injury is not very reliable in forecasting GUIs, not even when the AIS score of the pelvic girdle injury is included [14,15,19]. 

Eidelmann and coworkers have tried to predict bladder injuries based on total ISS alone and demonstrated a higher probability of bladder injuries in patients with an ISS>34 [7]. This cutoff proved feasible in our study as well, as patients with an ISS >34 were significantly more likely to sustain GUI (*p* = 0.032). While the ISS is an easy-to-use and world-renowned scoring system, its weaknesses, such as the clumsy division of six body regions, have been widely discussed [20,21,22]. Therefore, it does not seem reasonable to rely solely on the ISS to predict GUIs in severely injured patients. 

Another highly significant predictive factor for GUI was the patient’s sex. Due to the exposed anatomical location of the male genitalia and urethra, men are known to sustain traumatic LUTI more frequently compared to women [3,11]. In addition, men are known to be less risk averse and more often involved in high-energy trauma, such as motorcycle accidents [23]. This fact is also well reflected in our results: GUIs were significantly more frequent when the trauma was due to a motorcycle accident (*p* < 0.001) compared to all other trauma mechanisms. 

Our study also showed that macrohematuria presenting already in the emergency department is another highly significant predictor of GUI. It has been well described as a predictive factor for GUI in the literature but was found to be insufficient as the only criterion, since many patients without macrohematuria still have urogenital injuries [5,6,7]. 

All of the above-mentioned factors are highly significant predictors of the presence of GUI. However, as previously reported, each by itself does not have a high clinical value in predicting GUI in severely injured patients. Yet, combining these factors has allowed us to introduce a novel scoring system that can predict GUI in polytrauma patients with a very high probability. This scoring system is an easy-to-use tool, which can be utilized in the pre-hospital setting as well as in the emergency room setting. For example, a male motorcyclist with a suspected high-grade/unstable pelvic ring fracture and visible macrohematuria on the scene, should be transported to a trauma center with available urologic consultation. In addition, this patient would benefit from an early specialist urological examination in order to make a diagnosis quickly and thus not delay therapy.

We were pleased to find that the proposed novel scoring system has a high negative predictive value, as low scores of the UPPS predict the absence of urogenital injuries very well. Nevertheless, a low score does not exclude urogenital injuries with certainty, so the individual expertise of the treating physician and correct imaging are essential. Validation of the novel scoring system is still pending, with larger study populations needed to achieve validation. Furthermore, a prospective study should be conducted in the emergency department in the near future to determine the practical value of the scoring system. 

Even though penetrating and blunt abdominal trauma warrant for significantly different courses of treatment, we did not exclude these cases as we believe this very small amount of cases will not change statistics with any significance. 

The limitations of our study should also be considered, for example that data were collected retrospectively, raising the possibility of incomplete or inaccurate data collection. Considering this potential limitation, we excluded patients with incomplete data. 

## 5. Conclusions

Male sex, severe injuries of the pelvic girdle, motorcycle accidents, a high ISS total score of >34, and macrohematuria combined have been shown to be excellent predictors of urogenital injuries in polytrauma patients. The newly introduced UPPS-score provides treating emergency physicians with an easy-to-use tool to assess the likelihood of the presence of a GUI at an early stage. This way, further diagnostic measures can be initiated in time. High UPPS scores should alert emergency physicians to the possibility of the presence of GUIs. Nevertheless, the UPPS score should always be considered in correlation to the clinical findings. In case of high scores, diagnostic measures should be extended, and a urology specialist should be consulted early on. 

## Figures and Tables

**Table 1:** Demographics and trauma specific data, comparison of participating hospitals.

Variable		Hospital 1 (*n* = 215)		Hospital 2 (*n* = 198)		Total Study Population (*n* = 413)	
		Mean ± SD		Mean ± SD		Mean ± SD	
**Age (years)**		49.3 ± 19.5		55.8 ± 20.8		52.4 ± 20.4	
		*n*	(%)	*n*	(%)	*n*	(%)
**Sex**	Female	63	(29.3)	56	(28.3)	119	(28.8)
	Male	152	(70.7)	142	(71.7)	294	(71.2)
		Mean ± SD		Mean ± SD		Mean ± SD	
**ASA**		1.6 ± 0.7		1.7 ± 0.8		1.7 ± 0.7	
		*n*	(%)	*n*	(%)	*n*	(%)
**Number of patients**		215	(100)	198	(100)	413	(100)
**Mechanism of** **injury**	Car/truck passenger	41	(19.1)	39	(19.7)	80	(19.4)
	Motorcyclist	37	(17.2)	33	(16.7)	70	(16.9)
	Bicycle rider	22	(10.2)	25	(12.7)	47	(11.4)
	Pedestrian	22	(10.2)	16	(8.1)	38	(9.2)
	High fall (≥3 m)	65	(30.2)	41	(20.7)	106	(25.7)
	Low fall (<3 m)	22	(10.2)	39	(19.7)	61	(14.8)
	Stabbing	-	-	-	-	-	-
	Gunshot	1	(0.5)	3	(1.5)	4	(1.0)
	Other blunt trauma	5	(2.3)	1	(0.5)	6	(1.5)
	Not determinable	-	-	1	(0.5)	1	(0.2)
		Mean ± SD		Mean ± SD		Mean ± SD	
**AIS score of urogenital injuries**		2.3 ± 1.1		2.5 ± 1.0		2.3 ± 1.1	
		*n*	(%)	*n*	(%)	*n*	(%)
**Urogenital injuries**		32	(14.9)	15	(7.6)	47	(11.4)
		Median (IQR)		Median (IQR)		Median (IQR)	
**ISS**		29 (22–38)		32.5 (22–43)		29 (22–41)	
		*n*	(%)	*n*	(%)	*n*	(%)
**Mortality**		27	(12.6)	34	(17.2)	61	(14.8)

The characteristics of the 413 patients included in this study are given. Patient data was acquired in Hospital 1 and Hospital 2. *n* = number; ASA = American Society of Anesthesiologists; ISS = Injury Severity Scale; SD = standard deviation; IQR = interquartile range.

**Table 2:** Comparison of patient specific data and outcome parameters in the subgroups with and without urogenital injuries.

Variable		Urogenital Injury (*n* = 47)		No Urogenital Injury (*n* = 366)		*p*-Value
		Mean ± SD		Mean ± SD		
**Age (years)**		48.9 ± 16.7		52.9 ± 20.8		0.224
**ASA**		1.5 ± 0.6		1.7 ± 0.7		0.063
**Length of stay ICU (days)**		13.4 ± 18.2		12.5 ± 16.8		0.668
**Length of stay hospital (days)**		32.2 ± 27.9		27.0 ± 24.5		0.184
		Median (IQR)		Median (IQR)		
**ISS**		35 (21–50)		29 (22–38.75)		**0.032**
		*n*	(%)	*n*	(%)	
**Sex**	female	6	(12.8)	113	(30.9)	-
	male	41	(87.2)	253	(69.1)	**0.010**
**Mechanism of** **injury**	Car/truck passenger	10	(19.2)	70	(21.3)	0.725
	Motorcyclist	19	(40.4)	51	(14.0)	**0.001**
	Bicycle rider	2	(4.3)	45	(12.3)	0.102
	Pedestrian	4	(8.5)	34	(9.3)	0.862
	High fall (≥3 m)	6	(12.8)	100	(27.4)	0.033
	Low fall (<3 m)	1	(2.1)	60	(16.4)	0.009
	Stabbing	-	-	-	-	-
	Gunshot	1	(2.1)	3	(0.8)	0.389
	Other blunt trauma	4	(8.5)	2	(0.5)	0.061
**Macrohematuria**		18	(38.3)	27	(1.9)	**0.001**
**Mortality**		5	(10.6)	56	(15.3)	0.514

Characteristics of the 413 patients included in this study are given in subgroup analysis with and without urogenital injuries. Bold lettering indicates highly significant, positive predictive factors for GUIs in our patient cohort. *n* = number; ASA = American Society of Anesthesiologists; ISS = Injury Severity Scale; SD = standard deviation; IQR = interquartile range.

**Table 3:** The UPPS Score—introducing a novel scoring system.

	Severity/Quality	Points Scored	*p*
**Sex**	male/female	1/0	0.010
**Motorcycle accident**	yes/no	2/0	<0.001
**Pelvic fracture**	(AIS 1/2/3/4/5 or 6)	0/2/3/4/5	<0.001
**Macrohematuria**	yes/no	2/0	<0.001
**ISS**	>34/≤34	1/0	0.023

The UPPS (Urogenital trauma in Polytrauma patients with Pelvic and/or Spinal injuries) score allows stratification of polytrauma patients regarding the risk of urogenital injuries. Five parameters (sex, motorcycle accident, pelvic fracture, macrohematuria, ISS) are assessed and enable the prediction of the occurrence or absences of GUI in polytrauma. Points are given depending on statistical significance. Therefore, more points are scored in highly significant factors with *p*-values of <10^−3^. Scores between 7 and 11 make the presence of GUI likely. With a score between 4 and 6, the risk of urogenital injuries is moderate and should always be considered. A score from 0 to 3 makes the prevalence of urogenital injuries highly unlikely. ISS: Injury Severity Score.

## Data Availability

The datasets used and/or analyzed in the current study are available from the corresponding author on reasonable request.
